# Mislabelling, misclassification, misinterpretation: A critical appraisal of mortality trends in Alzheimer's and ischemic heart disease

**DOI:** 10.1016/j.ijcrp.2025.200464

**Published:** 2025-07-11

**Authors:** Hammad Jehangir

**Affiliations:** Department of Medicine, Allama Iqbal Medical College, Jinnah Hospital, Lahore, Pakistan

The article "Trends in mortality due to ischemic heart diseases among patients with Alzheimer's disease in the United States from 1999 to 2020″ by Akhtar et al. [1] tackles a timely and relevant question. However, it stumbles on multiple fronts: mislabelling metrics, misclassifying diagnoses, and misunderstanding statistics. With tighter editing and corrected methodology, it could become a meaningful contribution.

To start with, I'd like to point out the elephant in Figs. 1, 2, 3 and 4: “AAMR per 100,000 deaths”—an oxymoronic phrase that fundamentally misunderstands the definition of age-adjusted mortality rates. AAMR is calculated per 100,000 population, not per 100,000 deaths [2]. Mortality rates based on *death counts* are a statistical dead end. If this misunderstanding made its way into the figures or calculations, the results might be built on sand.

**Fig. 1:**
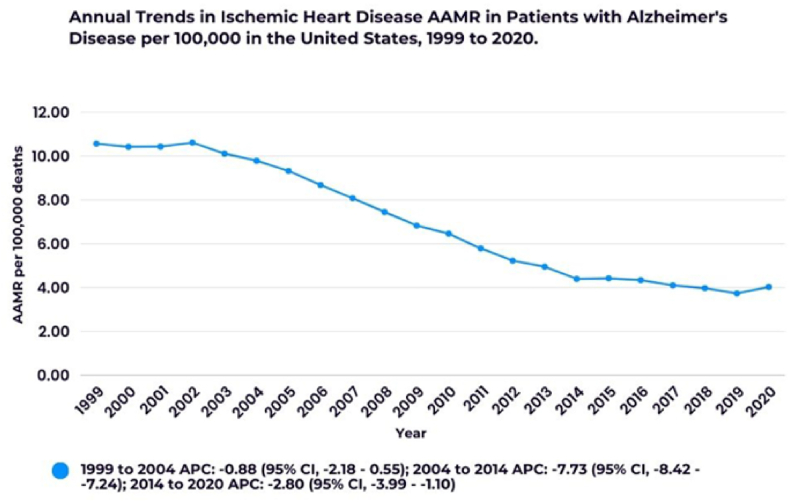
Annual trends in Ischemic Heart Disease AAMR in patients with Alzheimer's Disease. ∗Indicates that the Annual Percent Change (APC) is significantly different from zero at the alpha = 0.05 level.

**Fig. 2:**
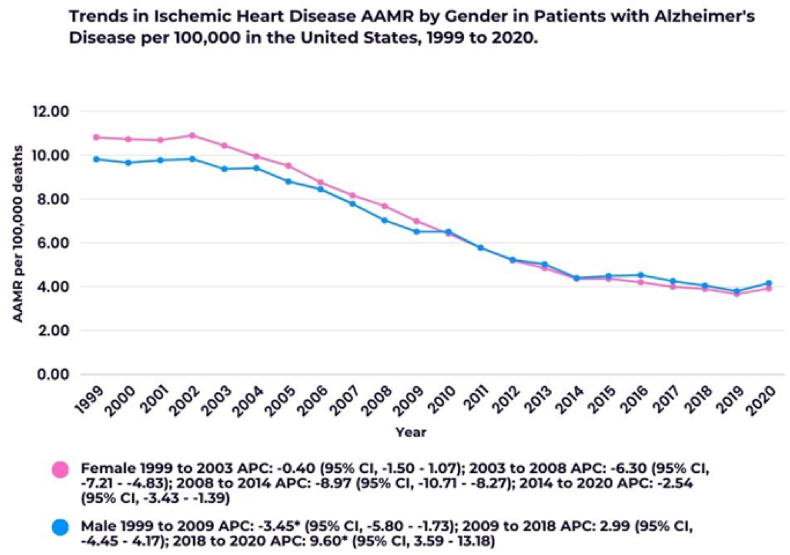
Annual trends in Ischemic Heart Disease AAMR in patients with Alzheimer's Disease stratified by sex. ∗Indicates that the Annual Percent Change (APC) is significantly different from zero at the alpha = 0.05 level.

**Fig. 3:**
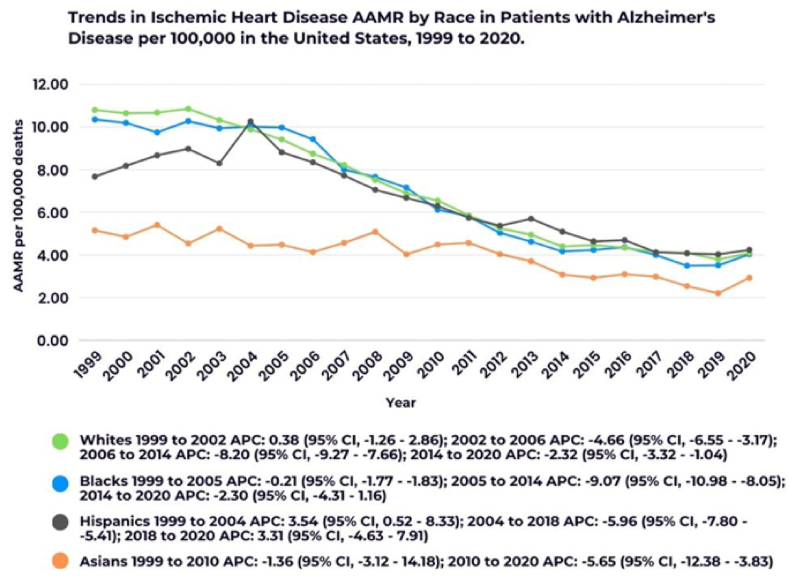
Annual trends in Ischemic Heart Disease AAMR in patients with Alzheimer's Disease stratified by race. ∗Indicates that the Annual Percent Change (APC) is significantly different from zero at the alpha = 0.05 level.

**Fig. 4:**
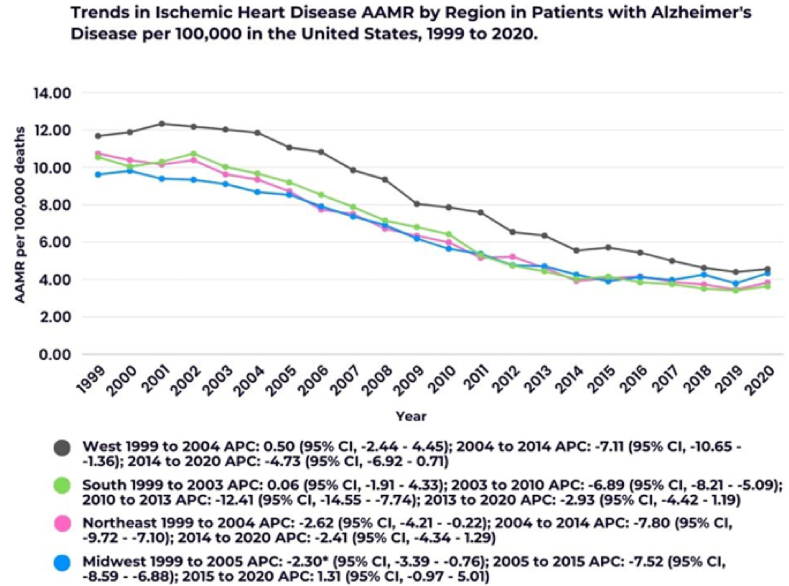
Annual trends in Ischemic Heart Disease AAMR in patients with Alzheimer's Disease stratified by regions. ∗Indicates that the Annual Percent Change (APC) is significantly different from zero at the alpha = 0.05 level.

The ICD-10 codes in the abstract contradict from those in the methods. Authors cite I25–I29 as ICD-10 codes for ischemic heart disease in the methods. That includes conditions like pulmonary embolism (I26) and various pulmonary heart and vessel diseases (I27–I28). Surprisingly, I29 *does* *not* *exist* on the CDC WONDER. The standard range for the relevant cohort is I20–I25 [3]. Misclassifying codes leads to misclassification bias and calls the entire cohort selection into question.

Throughout the results, the authors discuss declines in mortality trends even when confidence intervals include zero (e.g., APC: −2.30; 95 % CI: −4.31 to 1.16). These are not statistically significant by conventional standards [4]. Presenting these as trends without clear disclaimers misleads the reader. An explicit mention of statistical significance or the lack thereof would leave no room for ambiguity.

Overall, the paper provides useful insights into shifting mortality trends in a vulnerable population. Addressing key issues in data interpretation, terminology, and statistical presentation would enhance its scientific impact and reliability.

## Declaration of competing interests

The authors declare that they have no known competing financial interests or personal relationships that could have appeared to influence the work reported in this paper.
